# Plasma methionine concentrations and incidence of hypermethioninemic encephalopathy during infancy in a large cohort of 36 patients with classical homocystinuria in the Republic of Ireland

**DOI:** 10.1002/jmd2.12029

**Published:** 2019-03-26

**Authors:** John Allen, Bronwyn Power, Aida Abedin, Orla Purcell, Ina Knerr, Ahmad Monavari

**Affiliations:** ^1^ National Centre for Inherited Metabolic Disorders Temple Street Children's University Hospital Dublin Ireland

**Keywords:** encephalopathy, homocystinuria, hypermethioninemia, hypermethioninemic encephalopathy, methionine

## Abstract

**Background:**

Classical homocystinuria is an autosomal recessive disorder caused by profound cystathionine β‐synthase deficiency. Its biochemical hallmarks are high concentrations of plasma homocyst(e)ine and methionine. Clinical manifestations include lens dislocation, developmental delay, skeletal anomalies, or thromboembolism. Limited literature exists outlining the risk of encephalopathy associated with hypermethioninemia presenting in children with classical homocystinuria.

**Aim:**

To assess the quality of metabolic control and plasma methionine concentrations in infancy in a cohort of 36 patients with classical homocystinuria in the Republic of Ireland.

**Methods:**

Review of biochemical and clinical data including neuroradiological results that are available for the first year of life in our patients diagnosed on newborn screening was performed with appropriate consent and ethical approval.

**Results and Discussion:**

Median total homocyst(e)ine and methionine plasma concentrations were 78 and 55 μmol/L, respectively. Methionine concentrations were significantly higher in neonates as opposed to older children. The highest methionine level identified was 1329 μmol/L in a child who presented clinically with encephalopathy. Elevated homocyst(e)ine and methionine levels are associated with significant morbidities. Therefore, prevention of complications requires prompt recognition and treatment. Chronic and acute complications may be encountered in patients with classical homocystinuria and plasma methionine concentrations pose an additional risk factor.

## 1 INTRODUCTION

Classical homocystinuria (Online Mendelian Inheritance in Man [OMIM] #236200) is an inherited autosomal recessive disorder, which was first described in the literature independently by Carson and Neill^1^ and Gerritsen et al.^2^ It is an inborn error of the metabolism of essential sulfur‐containing amino acid methionine caused by a profound deficiency of cystathionine β‐synthase (CBS). The gene for this condition is located on chromosome 21 at 21q22.3. CBS deficiency leads to the accumulation of homocysteine in both blood and urine along with the oxidized form, homocystine. The term homocyst(e)ine may be used to refer to the total pool of homocysteine and homocystine.

Methionine can be regenerated from homocysteine by the action of methionine synthase with cobalamin as a cofactor. The true incidence of classical homocystinuria is unknown but varies worldwide between 1 in 1800 and 1 in 900 000.^3^ However, in Ireland, the introduction of the newborn bloodspot screening for classical homocystinuria in 1971 showed an incidence of 1:65 000.^4^

The clinical manifestations of untreated classical homocystinuria are multisystemic, including intellectual disability, a marfanoid habitus, osteoporosis, ectopia lentis, and severe myopia as well as thromboembolic phenomena. Clinical variability in the phenotypic features of homocystinuria is well‐recognized in the literature.^5^ Biochemical features include, in particular, increased plasma homocyst(e)ine (free and total homocyst(e)ine) and methionine concentrations as well as urinary excretion of homocystine. Pyridoxine, a cofactor for CBS, aids the conversion of homocysteine to cysteine via the production of cystathionine. A range of phenotypes of the disorder exist from a milder pyridoxine (vitamin B6)‐responsive form through to a more severe pyridoxine nonresponsive form. Life‐long treatment to lower plasma total homocyst(e)ine levels is required, which may include pyridoxine, a low methionine diet, a methionine‐free amino acid supplement, and sufficient supply of vitamin B12 and folic acid. Medications, such as betaine, should be considered as an adjunctive treatment in those who cannot achieve adequate control by other means alone. Betaine functions by donating a methyl group, thus converting homocysteine to methionine.^3^

There is limited literature outlining the rare occurrence of hypermethioninemic encephalopathy presenting in children with classical homocystinuria. Harvey Mudd et al.^6^ reported 10 cases of hypermethioninemia due to high methionine intake, and two of these infants had cerebral edema coinciding with extreme hypermethioninemia which resolved when methionine levels decreased.^6^ Lawson‐Yuen and Levy^7^ describe the occurrence of cerebral edema while using betaine to treat homocystinuria patients. Cerebral edema is reported to occur when plasma methionine exceeds, for example, 1000 μmol/L.^7^ Furthermore, central nervous system abnormalities have been reported in patients with methionine levels greater than 800 μmol/L although this was reported in patients with methionine adenosyltransferase I/III deficiency.^8^

We here assess the quality of metabolic control, including plasma homocyst(e)ine and methionine concentrations during infancy, in our cohort of 36 pediatric patients with classical homocystinuria in Ireland and the frequency of hypermethioninemia (ie, plasma methionine ≥600 μmol/L) with associated acute encephalopathy.

## 2 METHODS

Approval for the study was obtained from the local ethics and research committee and appropriate consent was sought and obtained prior to data collection. From our classical homocystinuria cohort diagnosed on newborn screening, we evaluated the results of biochemical profiles during infancy together with documented clinical findings in case notes and radiological imaging where available. Free and total homocyst(e)ine, cysteine, and methionine concentrations at ages 1 to 3 months, and at 6, 9, and 12 months of age were analyzed, while for the youngest patient only the first set of data was available. These time points were chosen as they represent the period at the time of establishment on a dietary regimen followed by weaning, alongside high metabolic demands, which can be a challenging time to maintain metabolic control. Due to the young age of the patients, samples were generally taken in a semi‐fasting state (ie, 4‐6 hours after their last feed) with occasional variations depending on waiting times for blood tests or other variables. Patients’ data were retrospectively analyzed and statistical comparisons between groups were performed with analysis of variance (ANOVA) using GraphPad Prism Software (GraphPad, Inc, San Diego, California).

## 3 RESULTS

A total of 36 patients with classical homocystinuria (17 male, 19 female) diagnosed on newborn bloodspot screening were analyzed. This represents our cohort of patients with classical homocystinuria, diagnosed since the introduction of newborn bloodspot screening in the Republic of Ireland in 1971. We did not include patients with a late/clinical diagnosis or patients born outside the Republic of Ireland. All patients presented here were commenced on a low‐protein diet after confirmation of a positive screening result for classical homocystinuria in an independent blood sample after their newborn bloodspot screening test came back positive and after a short‐trial period of pyridoxine.^4^ None of the patients presented here were classified as pyridoxine responsive. The average chronological age of these patients at time of this study was 26.6 years (0.5‐43.6 years). Free homocyst(e)ine, total homocyst(e)ine, methionine, and cystine plasma concentrations during infancy, that is, 1 to 3, 6, 9, and 12 months, were analyzed and the results are shown in Table 1.

**Table 1:** Concentrations of plasma amino acids (μmol/L) during infancy at four different age brackets in our cohort of 36 patients with classical homocystinuria

	Free homocyst(e)ine median (range)	Total homocyst(e)ine median (range)	Methionine median (range)	Cystine median (range)
Age 1‐3 months	9.5 (0‐45)	95 (5‐150)	70.5 (7‐1329)	34 (11‐180)
Age 6 months	8 (0‐52)	76 (13‐131)	59.5 (3‐314)	28 (10‐144)
Age 9 months	7 (0‐32)	77 (6‐184)	46.5 (14‐178)	28.5 (12‐163)
Age 12 months	10 (0‐36)	79 (57‐157)	50 (30‐136)	29 (13‐161)
Overall	8.75	78	54.75	28.75

**Figure 1:**
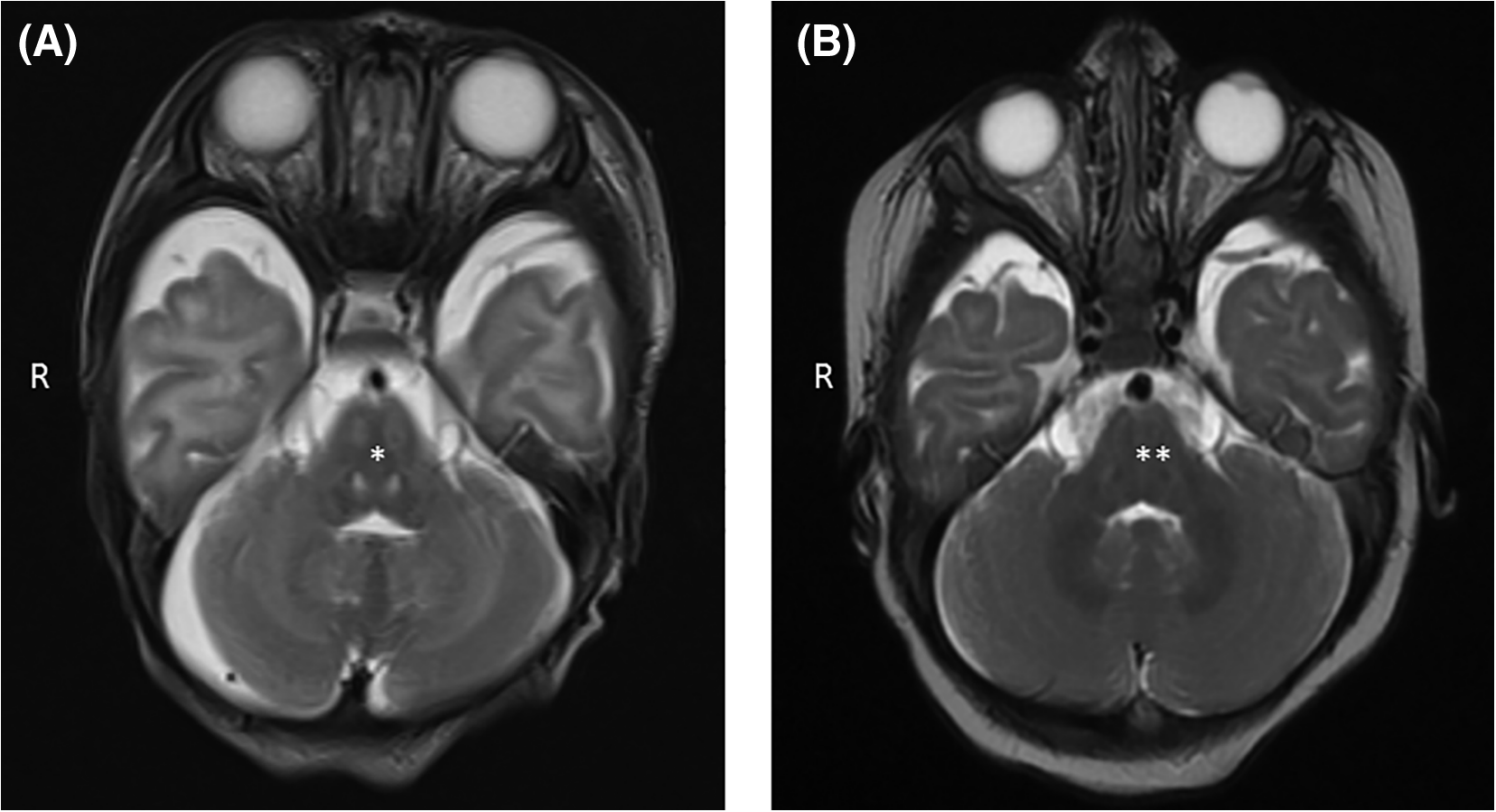
Magnetic resonance imaging brain in a 6‐week‐old baby with hypermethioninemic encephalopathy showing increased T2 signal intensity in the pons (A, *) and normalization 4 months later (B, **)

According to our policy, target total homocyst(e)ine plasma concentrations in patients with classical homocystinuria are <100 μmol/L and free homocyst(e)ine plasma levels <11 μmol/L. We found that the homocyst(e)ine (free/total) levels in our homocystinuria population were generally well‐controlled. Although there were no statistically significant differences for homocyst(e)ine (free/total) through the four different age brackets which we analyzed, methionine levels were significantly higher at 1 to 3 months of age than in older infants (ANOVA, *P* < 0.01) but there were no significant differences between the later time points. At age 3 months, the majority of patients would have been on a diet for approximately 2 months. However, we think that the slightly higher methionine concentrations at this time period, compared to the other age groups studied here may be due to relatively high‐protein intake (g/kg) and also recent adjustments of the patients' dietetic regimen. We were unable to analyze methionine concentrations in healthy individuals for comparison purposes. Pearson correlation analysis between total homocyst(e)ine and methionine levels showed a positive association (*r* = 0.4269, *P* < 0.01). The highest methionine level seen in the period in question was 1329 μmol/L in an infant who clinically presented with acute encephalopathy. This boy, our youngest patient at the time of the study, was diagnosed on day 4 of life with classical homocystinuria. His initial methionine level was 797 μmol/L (reference 5‐77 μmol/L) with a normal cystine of 27 μmol/L (reference 27‐92 μmol/L) and a raised total homocyst(e)ine of 131 μmol/L (reference <8 μmol/L) along with a free homocyst(e)ine of 19 μmol/L (normally undetectable). He was treated with vitamin supplements (pyridoxine 50 mg TDS and folic acid 5 mg OD) and a moderate protein restriction with 1 g of natural protein per kg per day along with a methionine‐free powdered infant formula to control his levels. He was found to be compound heterozygous for two pathogenic mutations in the *CBS* gene (c.919G>A and c.738delG). After 4 weeks of age, the boy presented with symptoms resembling an intercurrent illness to the local hospital. He was noted to have vomiting, poor oral feeding, and lethargy. He was commenced on IV antibiotics but a bacterial source of infection could not be established. Hyponatremia was noted (Sodium 126, Chloride 93 mmol/L). He was also noted to have hypertension (BP 125/66) and proteinuria. His renal ultrasound revealed increased echogenicity bilaterally with no evidence of renal vein thrombosis. His brain magnetic resonance imaging (MRI) performed within the first week of his admission to hospital showed areas of diffusion restriction extending inferiorly from the ventral posterolateral nucleus of the thalami bilaterally through the mid brain, pons, and also areas of less intense diffusion restriction within the corticospinal and corticobulbar tracts (Figure 1A). His MR spectroscopy and MR angiogram were normal. Intensive care treatment was required, including intubation and ventilation, and antihypertensive treatment with sodium nitroprusside followed by esmolol along with a carefully adjusted infusion regimen. No underlying infectious or thrombo‐embolic cause could be established. Overall, it was felt that his clinical presentation was metabolic in origin. His blood tests which were taken at the time of his acute presentation to his local hospital revealed a methionine of 1329 μmol/L with a normal cystine (23 μmol/L) and a raised total homocyst(e)ine (118 μmol/L) and free homocyst(e)ine (11 μmol/L). These levels improved promptly with increased calorie intake and anabolism, including intravenous dextrose, and further natural protein restriction. The patient made a full recovery; his brain MRI findings normalized (Figure 1B).

There was no other episode of symptomatic hypermethioninemic encephalopathy identified in our cohort. The second highest methionine level in our patient cohort was 600 μmol/L, which was noted in a female infant at age 3 months on a routine blood test. Free homocyst(e)ine was raised at 45 μmol/L. This infant did not have any neurological symptoms at the time and was clinically well. Treatment included increased calorie supply and further restriction of natural protein intake with follow‐up blood tests.

In this case of classical homocystinuria presented here, we identified that hypermethioninemic encephalopathy was characterized by the following features: substantial protein intake for a few days, followed by symptoms resembling an intercurrent illness such as vomiting and also progressive neurological deterioration; the patient had a pyridoxine‐nonresponsive form. It was established that although the patient was on a prescribed diet he was taking approximately 2.3 g/kg/day of natural protein for a number of days; it was not possible to ascertain why his prescribed formula was temporarily not available or not implemented. In the patient presented here with plasma methionine of 1329 μmol/L, associated biochemical features comprised mild hyponatremia and total homocyst(e)ine >100 μmol/L with an increased thromboembolic risk. MRI brain findings included areas of restricted diffusion throughout the midbrain and pons, and within the corticospinal and corticobulbar tracts. Clinically, intensive care management was required but the patient made a full recovery. We here demonstrate an incidence rate of one episode of symptomatic hypermethioninemic encephalopathy in 36 patient‐years. We did not identify any other homocystinuria‐associated complications in our cohort during infancy, such as thromboembolic events, through the four different age brackets, which we analyzed as part of this study.

## 4 DISCUSSION

The incidence of classical homocystinuria has been found to be higher in the Irish population than that which is reported worldwide due to the high prevalence (71%) of the pyridoxine‐nonresponsive cystathionine beta‐synthase mutation c.919G>A (p.G307S) but also other pathogenic variants.^9^ In our National Centre in Dublin, we studied 36 patients who have been diagnosed with classical homocystinuria since newborn screening for the condition began in the Republic of Ireland in 1971. We recently reported on our large cohort of patients diagnosed with classical homocystinuria on newborn screening and showed them to have good metabolic control.^10^ Here, we focused on four time‐points during the first year of life, including age 1 to 3, 6, 9, and 12 months, as this comprises the establishment of a dietetic regimen followed by weaning which may be challenging in terms of maintaining metabolic control. Overall, we found that plasma homocyst(e)ine levels (free/total) were generally well‐controlled in these age groups in our cohort over the years. However, methionine levels were significantly higher at age 1 to 3 months than in older infants. This was independent of homocyst(e)ine levels which were not significantly different throughout infancy. We, therefore, suggest that methionine should be considered to be a risk factor for the development of acute complications independent of homocyst(e)ine levels even in patients on established regimens. Although, much of the treatment in classical homocystinuria is aimed at preventing long‐term complications, physicians should be mindful of acute complications such as thromboembolic phenomena and rarely hypermethioninemic encephalopathy (one episode of hypermethioninemic encephalopathy in 36 patient‐years).

Encephalopathy is a term for a diffuse disorder of the brain that causes changes in function or structure. The causes for acute encephalopathy are numerous but may include intoxication, metabolic dysfunction, infection, electrolyte disturbances, hypertension, raised intracranial pressure, hypoxia, or a combination of these. Features of encephalopathy include altered mental status, lethargy, abnormal movements, nystagmus, dysphasia/aphasia, dysphagia, and seizures. Hypermethioninemic encephalopathy and its clinical consequences are potentially reversible with prompt diagnosis and treatment. However, failure to recognize encephalopathy can lead to permanent and irreversible brain abnormalities and can even be fatal.

Hypermethioninemia is known to cause metabolic encephalopathy. Harvey Mudd et al report 10 cases of hypermethioninemia associated with high methionine intake due to ingestion of a protein hydrolysate formula, which has now been removed from the market^6^; none of the patients in their study had homocystinuria. However, it is interesting to note that two of the infants described had MRI brain abnormalities indicative of cerebral edema at the time when their hypermethioninemia was most severe. These abnormalities resolved as methionine concentrations returned to normal. The pathogenesis of how hypermethioninemia could cause encephalopathy is incompletely understood. Harvey Mudd et al, in the same study (2003), noted brain‐imaging abnormalities that resembled cerebral edema, most marked in the cerebral cortex and posterior brainstem; the latter is similar to our findings. Harvey Mudd et al.^6^ put forward the hypothesis that Na(+),K(+)‐ATPase is inhibited by methionine and related metabolites.

Methionine concentrations may be elevated in individuals with classical homocystinuria whereas cysteine concentrations tend to be low which may be due to the defect in the metabolic pathway among other reasons, such as different protein binding characteristics or analytical methods. However, methionine plasma concentrations rarely reach levels sufficient to cause acute encephalopathy in patients on a protein‐restricted diet. This can be seen in patients on betaine therapy, for example, as discussed in Lawson‐Yuen and Levy.^7^ They found three cases reported in the literature of cerebral edema in association with hypermethioninemia in patients with classical homocystinuria on treatment with betaine.^11^, ^12^, ^13^

Betaine is an alternative methyl donor agent and lowers homocyst(e)ine in plasma through remethylation of homocysteine to methionine; it has been shown to be beneficial as an adjunctive therapy in preventing the complications of classical homocystinuria along with a 25% decrease in plasma homocyst(e)ine concentrations and 2‐ to 4‐fold rise in plasma methionine concentrations.^14^ The accompanying risk of a rise in plasma methionine is the reason why some metabolic centers, including our National Centre, use betaine predominantly for patients in whom sufficient metabolic control cannot be achieved with dietetic means and usually not in young infants. In some cases, plasma methionine can reach levels high enough to cause encephalopathy. Lawson‐Yuen and Levy report that encephalopathy can be seen in those with methionine levels greater than 1000 μmol/L, while Chien et al. have more recently described CNS abnormalities associated with methionine greater than 800 μmol/L.^8^ We have not found any publications describing hypermethioninemic encephalopathy in patients with classical homocystinuria who are not receiving treatment with betaine. By retrospective review of their biochemical results, we have shown that one of our patients with classical homocystinuria who was not on betaine treatment developed hypermethioninemia associated with encephalopathy. Taken together, we report a single patient with very high methionine concentrations who was not on treatment with betaine, and 35 patients without this complication. We also confirmed that none of our other patients with classical homocystinuria has developed clinical features of acute encephalopathy. It is reasonable to suggest, therefore, that this is a rare occurrence in pediatric patients with classical homocystinuria as this is hitherto the largest cohort‐study of patients with classical homocystinuria which addresses this question. Methionine should be monitored regularly in patients with classical homocystinuria, in particular during any episodes of acute deterioration.

## 5 CONCLUSIONS

Classical homocystinuria has a higher incidence in the Irish population as reported worldwide. Generally, a good metabolic control can be achieved in infants with a low‐methionine diet and medication and is important in preventing both the short‐term and long‐term complications of the condition. Hypermethioninemic encephalopathies are rarely seen in pediatric patients; however, excessively raised methionine concentrations can potentially cause acute metabolic encephalopathy with classical homocystinuria on treatment. Neuropsychiatric comorbidity is often recognized in patients with a late diagnosis of classical homocystinuria but acute complications due excessively raised methionine concentrations, that is, hypermethioninemic encephalopathy, may still be encountered even in individuals diagnosed on newborn screening on treatment, as demonstrated here. Therefore, further research into advanced treatment strategies is warranted for example, with a focus on enhanced clearance of homocyst(e)ine from the system, functional restoration of CBS, and prevention of endogenous toxicity.

## CONFLICT OF INTEREST

None.

## AUTHOR CONTRIBUTIONS

John Allen contributed to the collection, analysis, and interpretation of data and writing of the article. Bronwyn Power contributed to data collection, interpretation, and writing of the article. Aida Abedin contributed to data interpretation and writing of the article. Orla Purcell contributed to data collection and interpretation and writing of the article. Ina Knerr and Ahmad Monavari contributed to the design of the study, data collection and analysis, data interpretation, and writing of the article.

## ETHICAL APPROVAL

Approval for the study was obtained from the local ethics and research committee. No animals were used in conducting this study.

## PATIENT CONSENT

Appropriate consent was sought and obtained prior to data collection.

## DATA AVAILABILITY

Original data is held in the Children's University Hospital, Temple St, Dublin 1, Ireland.
